# Compressed glassy carbon maintaining graphite-like structure with linkage formation between graphene layers

**DOI:** 10.1038/s41598-019-43954-5

**Published:** 2019-05-17

**Authors:** Yuki Shibazaki, Yoshio Kono, Guoyin Shen

**Affiliations:** 1 Frontier Research Institute for Interdisciplinary Sciences, Tohoku University, 6-3 Aoba, Aramaki, Aoba-ku, 980-8578 Sendai, Japan; 2 International Center for Young Scientists, National Institute for Materials Science, 1-1 Namiki, 305-0044 Tsukuba, Ibaraki Japan; 3 HPCAT, Geophysical Laboratory, Carnegie Institution of Washington, 9700 South Cass Avenue, Argonne, 60439 Illinois, USA; 4 Present Address: Geodynamics Research Center, Ehime University, 2-5 Bunkyo-cho, 790-8577 Matsuyama, Ehime Japan; 5 Present Address: X-ray Science Division, Argonne National Laboratory, IL 60439 Argonne, USA

**Keywords:** Solid Earth sciences, Structure of solids and liquids

## Abstract

Amorphous diamond, formed by high-pressure compression of glassy carbon, is of interests for new carbon materials with unique properties such as high compressive strength. Previous studies attributed the ultrahigh strength of the compressed glassy carbon to structural transformation from graphite-like *sp*^2^-bonded structure to diamond-like *sp*^3^-bonded structure. However, there is no direct experimental determination of the bond structure of the compressed glassy carbon, because of experimental challenges. Here we succeeded to experimentally determine pair distribution functions of a glassy carbon at ultrahigh pressures up to 49.0 GPa by utilizing our recently developed double-stage large volume cell. Our results show that the C-C-C bond angle in the glassy carbon remains close to 120°, which is the ideal angle for the *sp*^2^-bonded honey-comb structure, up to 49.0 GPa. Our data clearly indicate that the glassy carbon maintains graphite-like structure up to 49.0 GPa. In contrast, graphene interlayer distance decreases sharply with increasing pressure, approaching values of the second neighbor C-C distance above 31.4 GPa. Linkages between the graphene layers may be formed with such a short distance, but not in the form of tetrahedral *sp*^3^ bond. The unique structure of the compressed glassy carbon may be the key to the ultrahigh strength.

## Introduction

Carbon is known to display numerous allotropes, such as graphite, diamond, fullerenes, carbon nanotubes, and glassy carbon, because of its flexibility to form chemical bonds with *sp*-, *sp*^2^-, and *sp*^3^-hybridizations. Glassy carbon (GC), which is an amorphous carbon allotrope, has attracted great interest in the fields of materials science, engineering, and industry, because of its unique physical and chemical properties such as low density, high-temperature stability, extreme resistance to chemical corrosion, and high impermeability to gases and liquids^1,2^. Two types of GCs are commercially available at the moment. A “low-temperature” GC, which is produced by heat treatment at ~1000–2000 °C (e.g., type I in Alfa Aesar, USA, and Sigradur K in HTW Hochtemperatur-Werkstoffe GmbH, Germany), consists of discrete fragments of distorted graphene layer^2–4^. A “high-temperature” GC produced at ~2500–3000 °C (e.g., type II in Alfa Aesar and Sigradur G in HTW Hochtemperatur-Werkstoffe GmbH) contains broken or imperfect fullerene-like nanospheroids encased in a disordered multilayer graphene matrix^2–4^. Both low- and high-temperature GCs are composed of nearly 100% *sp*^2^-bond at ambient condition^2^.

Recently, formations of superhard amorphous carbon have been found by high-pressure compression of type-I GC^5–8^. When a type-I GC is compressed above ~30–40 GPa under room temperature, it has been reported to possess high compressive strength comparable to or higher than that of the crystalline diamond^5,6^, with the compressed GC referred to as an “amorphous diamond”. Furthermore, by heat treatment on the compressed GC under high-pressure conditions, the superhard GCs can be recovered to ambient condition^7,8^, implying the potential of this material for wide engineering applications. The superhard GC recovered to ambient condition was named a “quenchable amorphous diamond”^8^.

The enhancements in mechanical properties of the compressed type-I GC have been explained by structural transformation of the type-I GC. *In situ* synchrotron X-ray Raman spectroscopy (XRS) measurement for the type-I GC showed a decrease of *π*-bonding feature with increasing pressure and its eventual disappearance at 44.4 GPa^5^. The disappearance of the *π*-bonding feature was interpreted as a transformation from a nearly 100% *sp*^2^-bonded graphite-like structure at ambient pressure to a fully *sp*^3^-bonded diamond-like structure above 44.4 GPa^5^. Similarly, optical Raman spectroscopy measurement for the type-I GC showed a weakening of the G-band (~1600 cm^−1^) feature at high pressures, which was also interpreted as the result of the *sp*^2^-*sp*^3^ structural transformation at high pressures^6^. In addition, it has been reported that GCs with partially^7^ or fully^8^ *sp*^3^-bonded structures were synthesized by heat treatments under high-pressure conditions (25 GPa and 400–1000 °C for the partial *sp*^3^-bonded structure^7^; 50 GPa and 1523 °C for the full *sp*^3^-bonded structure^8^). Electron energy loss spectroscopy (EELS) measurements of these recovered samples showed reduction or disappearance of the *π*-bonding feature, which was also considered as the evidence of partially^7^ or fully^8^ *sp*^3^-bonded structure in the compressed type-I GC. For the “high-temperature” GC, recent studies^9,10^ on high-pressure structural behavior of a Sigradur-G GC showed that the *sp*^3^ bond drastically increases above 30 GPa under cold-compression, associated with collapses of fullerene-like structures.

However, all the previous studies on the compressed GC show only a decrease or disappearance of the *π*-bonding feature as evidence of the transformation from the graphite-like *sp*^2^-bonded structure to the diamond-like *sp*^3^-bonded structure. Although the disappearance of the *π*-bonding feature may indicate some bonding changes in the compressed GC, it does not directly indicate the formation of the diamond-like *sp*^3^-bonded structure. In addition to the EELS measurement, Zeng *et al*.^8^ measured X-ray diffraction (XRD) patterns of the quenched amorphous diamond, and compared the measured data with the structure factor of a fully tetrahedral *sp*^3^-bonded amorphous carbon structure from ab initio molecular dynamics simulations. It is noticeable that the first and the second peak positions of the XRD pattern of the quenched amorphous diamond are different from the simulation result. It is still unclear whether the so-called “amorphous diamond” consists of the diamond-like *sp*^3^-bonded structure.

To identify the bonding state in the compressed GC (i.e., *sp*^2^- or *sp*^3^-bonded structure), the C-C-C bond angle obtained from the pair distribution functions *g*(*r*) is the most essential information. In the *sp*^2^-bonded structure such as graphite, the C-C bond structure consists of trigonal planar geometry with the C-C-C bond angle of 120°. On the other hand, in the *sp*^3^-bonded structure such as diamond, a tetrahedrally coordinated carbon exhibits the C-C-C bond angle of 109.5°. Thus the C-C-C bond angle can provide a direct and critical test for the *sp*^2^- or *sp*^3^-bonded structure under high pressures. The *g*(*r*) and the C-C-C bond angle of a GC at high pressures have been experimentally determined by Zhao *et al*.^4^, whereas the experimental pressure was limited to 14.3 GPa, because of experimental challenges such as requirement of large sample volume for measuring X-ray diffraction from weakly scattering GC samples.

In this paper, we determined *g*(*r*) of the type-I GC at high-pressure conditions up to 49.0 GPa under room temperature by utilizing our recently developed double-stage large volume cell^11,12^ combined with a multi-angle energy dispersive X-ray diffraction (EDXD) technique^13^. The double-stage large volume cell enables us to compress large volume sample of 0.4 mm in diameter and 0.19 mm in height, which is approximately 10^3^ times larger than a typical sample volume in the diamond anvil cell^8^, to high pressure conditions to 49.0 GPa. Such large sample volume is crucial for determining *g*(*r*) reliably for the low scattering material of GC. We found that the C-C-C bond angle in the GC slightly decreases with increasing pressure below 17.7 GPa, while it remains at ~120° up to 49.0 GPa. It clearly indicates that the type-I GC mainly consists of graphite-like structure up to 49.0 GPa, and does not transform to diamond-like tetrahedral *sp*^3^-bonded structure. On the other hand, the interlayer distance in the GC, i.e., the distance between graphene layers, significantly decreases with increasing pressure, and eventually approaches to the second neighbor C-C distance in graphene fragments at 31.4 GPa. Such a short distance between graphene layers may form linkages between the layers, which may be responsible for the high compressive strength of the compressed GC reported in previous studies^5,6^.

## Results and Discussion

**Figure 1:**
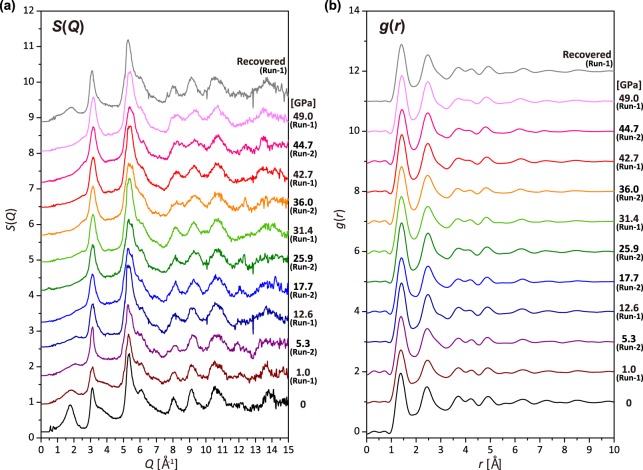
Structure factors *S*(*Q*) and pair distributuion functions *g*(*r*) of a glassy carbon (GC). (**a**) *S*(*Q*) of GC up to 49.0 GPa. The *S*(*Q*) for both Run-1 (1.0, 12.6, 31.4, 42.7, and 49.0 GPa) and Run-2 (5.3, 17.7, 25.9, 36.0, and 44.7 GPa) are shown together. The *S*(*Q*) of recovered sample from 49.0 GPa from Run-1 is also shown. (**b**) *g*(*r*) of GC obtained from *S*(*Q*) up to 49.0 GPa and after decompression. *S*(*Q*) and *g*(*r*) are displayed by a vertical offset of 0.8 and 1.5, respectively.

**Figure 2:**
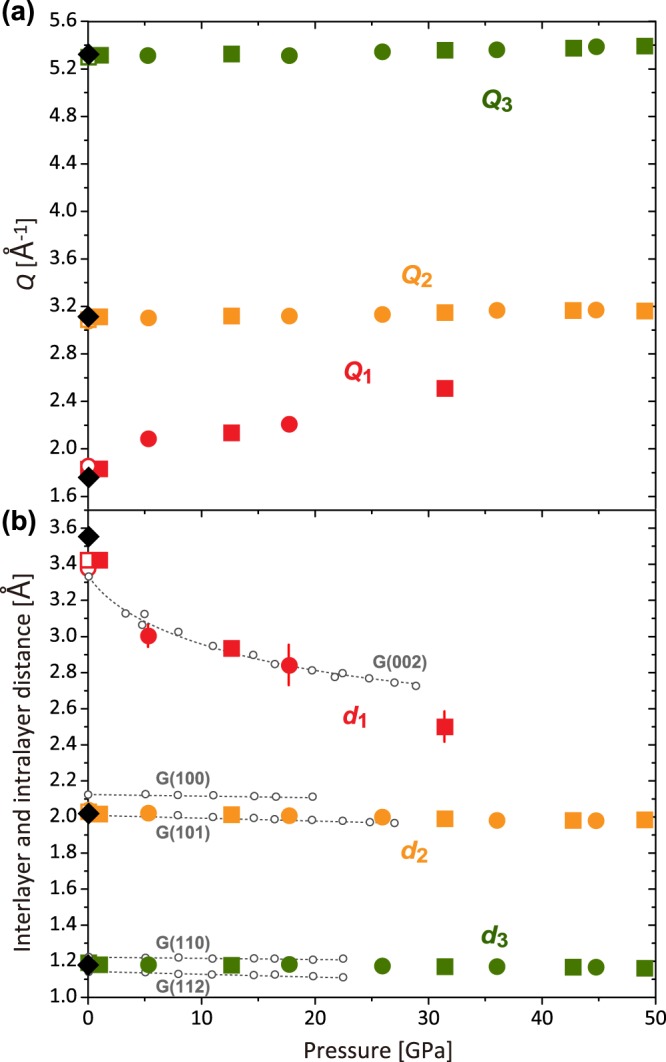
Pressure dependences of peak positions of *S*(*Q*) and corresponding interlayer and intralayer distances. (**a**) The first (*Q*_1_) (red), second (*Q*_2_) (orange), and third (*Q*_3_) (green) peak positions of *S*(*Q*). Square and circle symbols denote the data from Run-1 and Run-2, respectively. Black diamonds and open symbols at 0 GPa denote the data of the starting material and the recovered samples for Run-1 (square) and Run-2 (circle), respectively. (**b**) Corresponding interlayer (*d*_1_) and intralayer (*d*_2_ and *d*_3_) distances. The *d*-spacings of (002), (100)/(101), and (110)/(112) of compressed graphite^14^ are plotted for comparison (open gray circles and dotted lines). Peak positions of the *S*(*Q*) are determined by fitting with Gaussian function. Errors in the peak positions are determined as three sigma of the standard deviation of the fitting. Most of the error bars are within the symbol sizes. The values of *Q*_1_, *Q*_2_, *Q*_3_, *d*_1_, *d*_2_, and *d*_3_ in this study are summarized in Table 1.

Figure 1 shows structure factors, *S*(*Q*), and pair distribution functions, *g*(*r*), of the type-I GC up to 49.0 GPa. The *S*(*Q*) at ambient pressure exhibits three intense peaks at *Q*_1_ = 1.77(1), *Q*_2_ = 3.12(1), and *Q*_3_ = 5.33(1) Å^−1^, corresponding to the real space distances of *d*_1_ = 3.56(1), *d*_2_ = 2.02(1), and *d*_3_ = 1.18(1) Å, respectively (*d* = 2*π*/*Q*) (Fig. 2). These features of *S*(*Q*) are the same as the previous results measured at low pressures up to 14.3 GPa^4^. The *d*_1_, *d*_2_, and *d*_3_ are comparable to the *d*-spacings of crystalline graphite of (002), (100)/(101), and (110)/(112), respectively (Fig. 2b). Therefore, the *d*_1_ corresponds to the interlayer distance of graphite-like layered structure, while the *d*_2_ and *d*_3_ correspond to the intralayer distances. The shoulder peaks at ~3.6 and ~6.2 Å^−1^ in *S*(*Q*) (Fig. 1a) are higher-order diffractions of the first and second intense peaks.

The interlayer distance (*d*_1_) considerably shortens with increasing pressure, compared to the intralayer distances (*d*_2_ and *d*_3_) (Fig. 2b). The compression behavior of the *d*_1_, *d*_2_, and *d*_3_ distances are in good agreement with those of the *d*-spacing of (002), (100)/(101), and (110)/(112) of compressed graphite^14^, respectively, indicating that the type-I GC compresses in the same manner as the graphite (Fig. 2b). The first peak of *S*(*Q*) markedly weakens with increasing pressure, and eventually disappears at pressures between 31.4 and 36.0 GPa (Fig. 1a). After releasing pressure, the first peak of *S*(*Q*) appears again (Fig. 1a).

**Figure 3:**
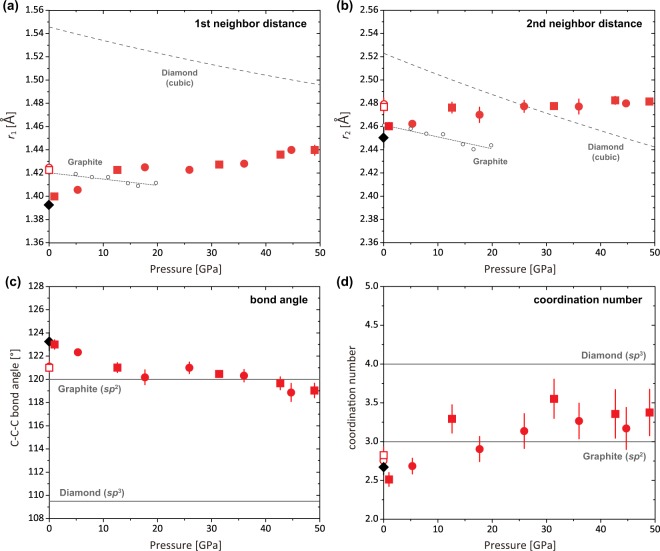
Pressure dependences of the average first and second neighbor C-C distances, average C-C-C bond angle, and coordination number. The first (*r*_1_) (**a**) and second (*r*_2_) (**b**) neigbor distances in GC. Square and circle symbols denote the data from Run-1 and Run-2, respectively. Black diamonds and open symbols at 0 GPa denote the data of the starting material and the recovered samples for Run-1 (square) and Run-2 (circle), respectively. Open gray circles and dotted lines denote the *r*_1_ and *r*_2_ distances of graphite calculated from the (100) *d*-spacing values of the graphite^14^ assuming the regular hexagonal carbon ring. Dashed lines denote the *r*_1_ and *r*_2_ distances of cubic diamond calculated from the volume data reported by Occelli *et al*.^19^. (**c**) The C-C-C bond angle calculated from *r*_1_ and *r*_2_ according to McKenzie *et al*.^15^. The ideal C-C-C bond angles of graphite (*sp*^2^-bonding), 120°, and diamond (*sp*^3^-bonding), 109.5°, are shown for comparison. (**d**) The coordination number (CN) calculated from the radial distribution function and number density of graphite^16^. The CN of graphite (*sp*^2^-bonding), CN = 3, and diamond (*sp*^3^-bonding), CN = 4, are shown for comparison. Peak positions of the *g*(*r*) are determined by fitting with Gaussian function. Errors in the peak positions are determined as three sigma of the standard deviation of the fitting. Most of the error bars are within the symbol sizes. The values of *r*_1_, *r*_2_, bond angle, and CN in this study are summarized in Table 1.

Figure 3a,b show the first (*r*_1_) and second (*r*_2_) peak positions of *g*(*r*), which correspond to the average first and second neighbor C-C distances of the GC, respectively. The average C-C-C bond angle (*θ*) is calculated from the *r*_1_ and *r*_2_ [*θ* = 2sin^−1^(*r*_2_/2*r*_1_)]^15^ (Fig. 3c), and the first coordination number (CN) is determined from the radial distribution function using the density data of the crystalline graphite^16^ (Fig. 3d). O’Malley *et al*.^17^ reported that the *S*(*Q*) obtained from X-ray scattering as this study represents the graphite-like short-to-medium range structure rather than micropores in the GC, because the micropores is at 50–100 Å scale^18^, and they proposed that the use of the density of graphite is suitable for the calculation of the CN of GC from the radial distribution function, rather than the bulk density of GC.

The *r*_1_ of 1.39(1) and *r*_2_ of 2.45(1) Å in GC at ambient pressure is comparable to those of graphite (1.42 and 2.46 Å, respectively). The *r*_1_ and *r*_2_ gradually increase with increasing pressure (Fig. 3a,b), and we observed the *r*_1_ and *r*_2_ distances of 1.44(1) and 2.48(1) Å, respectively, at the highest pressure of 49.0 GPa. The *r*_1_ distance of the compressed GC at 49.0 GPa is markedly smaller than that of diamond^19^ (1.497 Å) (Fig. 3a). The C-C-C bond angle at ambient pressure is 123.3(8)°, which is slightly larger than the ideal C-C-C bond angle in a carbon six-membered ring, 120°. At high pressures, the C-C-C bond angle decreases with increasing pressure at 0–17.7 GPa and it becomes almost constant at ~120(2)° above 17.7 GPa (Fig. 3c). In addition, the CN of 2.67(9) at ambient pressure is slightly lower than that of graphite (CN = 3). The CN increases with increasing pressure and it becomes almost constant around 3–3.5 at high pressures (Fig. 3d).

Our observed C-C-C bond angles close to 120° up to 49.0 GPa clearly indicate that the graphene layers are largely intact. Although previous spectroscopic studies^5,6^ suggested a *sp*^2^-*sp*^3^ transformation in the type-I GC at 30–40 GPa, our results of the compressed GC up to 49.0 GPa are inconsistent with those of diamond-like structure that would display an average C-C-C bond angle of ~109.5°. Therefore, the so-called “amorphous diamond” is an inappropriate term for the superhard compressed GCs from the structure point of view.

We observed that the average interlayer distance (*d*_1_) of the compressed GC decreases sharply with increasing pressure, and becomes very small at high pressure conditions (Fig. 2b); the *d*_1_ at 31.4 GPa [2.50(9) Å] becomes comparable to the second neighbor C-C distance (*r*_2_) of 2.48(1) Å (Table 1), and it is expected to shorten further at higher pressures. The significant shortening of the interlayer distance at high pressure conditions may enhance interaction between graphene layers and eventually form linkages between the graphene layers as suggested by Hu *et al*.^7^. Similarly to our results, Hu *et al*.^7^ showed reduction of the interlayer distance in the type-I GC after high-pressure and high-temperature treatment, and proposed the formation of *σ*-bond bridging at locally curved or buckling surfaces of the graphene layers. Our data show that the average CN of the nearest neighbor C-C bond distance increases with increasing pressure and becomes larger than three (Fig. 3d), which may suggest formation of interlayer linkages at high pressure conditions.

**Table 1:** Summary of the determined experimental parameters: the first (*Q*_1_), second (*Q*_2_), and third (*Q*_3_) peak positions of *S*(*Q*), the corresponding interlayer (*d*_1_) and intralayer (*d*_2_ and *d*_3_) distances, the first (*r*_1_) and second (*r*_2_) neighbor C-C distances, the calculated C-C-C bond angles, and the coordination number (CN).

Pressure [GPa]*	*Q*_1_ [Å^−1^]	*d*_1_ [Å]	*Q*_2_ [Å^−1^]	*d*_2_ [Å]	*Q*_3_ [Å^−1^]	*d*_3_ [Å]	*r*_1_ [Å]	*r*_2_ [Å]	Angle [°]	CN
0	1.77 (1)	3.56 (1)	3.12 (1)	2.02 (1)	5.33 (1)	1.18 (1)	1.39 (1)	2.45 (1)	123.3 (8)	2.67 (9)
**Run-1**										
1.0 (2)	1.83 (1)	3.43 (2)	3.11 (1)	2.02 (2)	5.32 (1)	1.18 (1)	1.40 (1)	2.46 (1)	123.0 (4)	2.51 (9)
12.6 (3)	2.14 (1)	2.94 (3)	3.12 (1)	2.01 (1)	5.33 (1)	1.18 (2)	1.42 (1)	2.48 (1)	121.0 (4)	3.29 (18)
31.4 (3)	2.51 (3)	2.50 (9)	3.15 (1)	1.99 (2)	5.36 (1)	1.17 (2)	1.43 (1)	2.48 (1)	120.5 (1)	3.55 (25)
42.7 (3)	—	—	3.17 (1)	1.98 (1)	5.38 (2)	1.17 (2)	1.44 (1)	2.48 (1)	119.7 (6)	3.36 (31)
49.0 (3)	—	—	3.17 (1)	1.98 (3)	5.40 (1)	1.16 (1)	1.44 (1)	2.48 (1)	119.0 (6)	3.38 (30)
Recovered	1.84 (1)	3.42 (3)	3.09 (1)	2.03 (1)	5.31 (1)	1.18 (1)	1.42 (1)	2.48 (1)	121.0 (4)	2.82 (10)
**Run-2**										
5.3 (1)	2.09 (2)	3.01 (6)	3.11 (1)	2.02 (1)	5.31 (1)	1.18 (1)	1.41 (1)	2.46 (1)	122.3 (3)	2.68 (10)
17.7 (3)	2.21 (4)	2.84 (11)	3.13 (1)	2.01 (1)	5.32 (1)	1.18 (1)	1.42 (1)	2.47 (1)	120.2 (6)	2.90 (16)
25.9 (5)	—	—	3.14 (1)	2.00 (2)	5.35 (2)	1.18 (2)	1.42 (1)	2.48 (1)	121.0 (5)	3.14 (22)
36.0 (6)	—	—	3.17 (1)	1.98 (3)	5.36 (1)	1.17 (2)	1.43 (1)	2.48 (1)	120.3 (5)	3.27 (23)
44.7 (2)	—	—	3.17 (1)	1.98 (2)	5.39 (1)	1.17 (2)	1.44 (1)	2.48 (1)	118.4 (8)	3.17 (27)
Recovered	1.86 (2)	3.38 (7)	3.08 (1)	2.04 (1)	5.31 (1)	1.18 (1)	1.42 (1)	2.48 (1)	121.1 (4)	2.75 (11)

*Pressure was measured before and after each structure measurement of the GC sample, because of the long measurement time (~9 hours), and the average pressures obtained before and after measurements are shown with the pressure differences as errors.

Such linkages between graphene layers may be responsible for the disappearance of *π*-bonding feature in the XRS carbon K-edge spectra in Lin *et al*.^5^ and the disappearance of G-band in the Raman spectra in Yao *et al*.^6^ at 30–40 GPa. However, considering our observed C-C-C bond angles of ~120° up to 49.0 GPa, the linkages do not mean to form the tetrahedral *sp*^3^-bonding of crystalline diamond-like structure proposed by previous studies^5,6^, but could be an anisotropic four-fold bonding between the trigonal planar layers. A recent study of Shiell *et al*.^10^ shows a calculation result of existence of two different bond lengths in the *sp*^3^ bond in the compressed GC, with the bonds aligned parallel to the compression axis being longer than those perpendicular to the compression axis, at high pressures, which also implies anisotropic four-fold bonding structure.

It is interesting to note that the *S*(*Q*) of the GC at >36.0 GPa measured in this study is similar to the XRD pattern of the “quenchable amorphous diamond” synthesized by Zeng *et al*.^8^ at 50 GPa and 1800 K, which showed two intense peaks at ~3.1 and ~5.3 Å^−1^ without first peak around ~1.7 Å^−1^ in the XRD pattern. Zeng *et al*.^8^ also showed the disappearance of the *π*-bonding feature from the EELS measurement of the quenched amorphous diamond, which is the same as the cold-compressed GC by Lin *et al*.^5^. These observations imply that the “quenchable amorphous diamond” may consist of the compressed graphite-like structure with linkages between the graphene layers proposed in this study, rather than 100% diamond-like tetrahedral *sp*^3^-bonded structure presented by Zeng *et al*.^8^. In addition, it has been reported that the crystalline graphite also loses half of the *π*-bonding feature at ~17 GPa at room temperature, based on inelastic X-ray scattering spectroscopy investigations^20^. Mao *et al*.^20^ also suggested that the cold-compressed graphite consists of distorted graphene layers with partially bridging between the layers by *σ* bonds. The structural model of the cold-compressed graphite by Mao *et al*.^20^ is similar to that of the compressed GCs in this study.

Moreover, the “high-temperature” GC (Sigradur-G) has been also reported to exhibit the *sp*^2^-*sp*^3^ transformation above 30 GPa^9,10^. It is known that the interlayer distance between disordered graphene layers in the “high-temperature” GC, which encase fullerene-like nanospheroids, decreases with increasing pressure in the same manner as that in the “low-temperature” GC such as type-I GC at least up to 14.3 GPa^4^. The *sp*^3^ bonds in the “high-temperature” GC suggested by Shiell *et al*.^9,10^ may also be the same as that of the compressed type-I GC proposed in this study. Further investigations such as precise *g*(*r*) of the “high-temperature” GC will be required to understand its bonding structure (e.g., diamond-like tetrahedral *sp*^3^-bonded structure or anisotropic four-fold bonded structure) at high pressures.

Our pair distribution function data revealed unique structure of the compressed glassy carbon with significantly short graphene interlayer distance and possible linkages between the interlayer, which may be the origin of the ultrahigh strength and hardness of the compressed GCs suggested by previous studies^5,6,8^. In fact, compressed GCs synthesized at 25 GPa with varying temperature by Hu *et al*.^7^ shows marked increase of compressive strength and hardness with reduction of graphene interlayer distance. Because of relatively low-pressure condition of the experiments by Hu *et al*.^7^, the average interlayer distance is still long (~2.7 Å at 25 GPa in Fig. 2b) and the strength and hardness are still lower than those of diamond. On the other hand, our data show that the graphene interlayer distance further decreases by further compression to 49.0 GPa, which is expected to increase compressive strength and hardness of compressed GC, possibly comparable to those of crystalline diamond as reported in some previous studies^5,6,8^. The formation of the linkages between the graphene layers could change not only the strength and hardness but also the electronic property^21^. Control of the interlayer distance in the GC by pressure would be a key to synthesizing various amorphous carbons with unique properties.

In summary, we have determined *g*(*r*) of the type-I GC up to 49.0 GPa under room temperature, by using the double-stage large volume cell combined with the multi-angle EDXD technique. Our results clearly show that the C-C-C bond angle stays at ~120° up to 49.0 GPa, and indicate that the type-I GC remains the graphite-like structure, instead of the transformation from graphite-like *sp*^2^-bonded structure to diamond-like tetrahedral *sp*^3^-bonded structure proposed by previous studies^5,6^. On the other hand, we observed that the interlayer distance between graphene layers significantly shortens under ultrahigh pressure conditions and it becomes almost comparable to the second neighbor C-C distance at 31.4 GPa. The short interlayer distance above 31.4 GPa may cause formation of the linkages between graphene layers, as shown by the disappearance of *π*-bonding feature in Lin *et al*.^5^. Our results propose that the compressed GCs consist of graphite-like layered structure with linkages between compacted graphene layers. The so-called “amorphous diamond” is inappropriate.

## Methods

### High-pressure experiments

**Figure 4:**
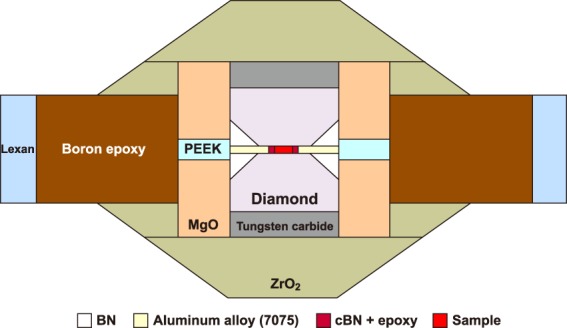
Illustration of cell design. We slightly modified the double-stage cell assembly of Kono *et al*.^11^. We used PEEK (polyether ether ketone) cylinder in the middle of the MgO sleeve and removed TiB_2_ sleeve for minimizing X-ray absorption through the cell for obtaining better X-ray signal from the weak X-ray scattering GC sample.

High-pressure experiments were conducted using the double-stage large volume cell^11,12^ at the beamline 16-BM-B, High Pressure Collaborative Access Team (HPCAT) in the Advanced Photon Source^13^. We slightly modified the double-stage cell assembly of Kono *et al*.^11^ (Fig. 4). We used a pair of (100)-oriented single crystal diamonds with a 1.2 mm culet as the second stage anvil. The large culet size of the second stage diamond anvil enables us to use large sample volume of 0.4 mm in diameter and 0.19 mm in height. We used a type-I GC plate (Alfa Aesar, 1.61 g/cm^3^ by Archimedes’ method) as the sample, which is the same material used in the reported studies^5–8^. The GC sample was placed in the 0.5 mm diameter gasket hole without pressure medium to avoid diffraction peaks from the pressure medium in the XRD measurement. Pressure was determined by the equation of state of Au^22^, placed at the edge of the sample. Recovered samples from high-pressure conditions display black in color and not optically transparent.

### Structure measurement by synchrotron X-ray diffraction

The structure measurements for GC were carried out via *in situ* multi-angle EDXD technique^13^. We conducted two high-pressure experiments (Table 1). The structure of GC at ambient pressure was separately measured. The size of the incident white X-ray was adjusted to 0.1 mm in the vertical direction by a slit and the white X-ray was focused to 0.009 mm (full width at half maximum) in the horizontal direction by a Pt-coated mirror^12^. We collected a series of EDXD patterns at 2*θ* angles of 4.1°, 5.1°, 6.6°, 8.1°, 10.6°, 13.6°, 17.1°, 21.1°, 27.1°, and 33.1° using a Ge solid state detector (Canberra). Primary structure factor result was derived from the observed EDXD patterns using a software package developed by Dr. Funakoshi^23^. Pair distribution function was obtained by the Fourier transformation of the structure factor with the maximum *Q* of 15.0 Å^−1^. We applied the Kaplow-type optimization procedure to determine final structure factor, *S*(*Q*), and pair distribution function, *g*(*r*), using an optimization procedure by Shen *et al*.^24^. The iteration in the optimization process is seven. The Lorch function was applied to remove the truncation effect on the final pair distribution function determination^25^.
